# A physiological glucocorticoid rhythm is an important regulator of brown adipose tissue function

**DOI:** 10.1016/j.molmet.2021.101179

**Published:** 2021-02-03

**Authors:** Jan Kroon, Maaike Schilperoort, Wietse In het Panhuis, Rosa van den Berg, Lotte van Doeselaar, Cristy R.C. Verzijl, Nikki van Trigt, Isabel M. Mol, Hetty H.C.M. Sips, Jose K. van den Heuvel, Lisa L. Koorneef, Ronald J. van der Sluis, Anna Fenzl, Florian W. Kiefer, Sabine Vettorazzi, Jan P. Tuckermann, Nienke R. Biermasz, Onno C. Meijer, Patrick C.N. Rensen, Sander Kooijman

**Affiliations:** 1 Department of Medicine, Division of Endocrinology, Leiden University Medical Center, Leiden, the Netherlands; 2 Einthoven Laboratory for Experimental Vascular Medicine, Leiden University Medical Center, Leiden, the Netherlands; 3 Department of Medicine, Division of Nephrology, Leiden University Medical Center, Leiden, the Netherlands; 4 Clinical Division of Endocrinology and Metabolism, Department of Medicine, Medical University of Vienna, Vienna, Austria; 5 Institute for Comparative Molecular Endocrinology, University of Ulm, Ulm, Germany; 6 Department of Endocrinology, the First Affiliated Hospital of Xi'an Jiaotong University, Xi'an Jiaotong University, Xi'an, China

**Keywords:** Brown adipose tissue, Circadian Rhythm, Corticosterone, Glucocorticoid receptor

## Abstract

**Objective:**

Brown adipose tissue (BAT) displays a strong circadian rhythm in metabolic activity, but it is unclear how this rhythm is regulated. As circulating levels of corticosterone coincide with the rhythm of triglyceride-derived fatty acid (FA) uptake by BAT, we investigated whether corticosterone regulates BAT circadian rhythm.

**Methods:**

Corticosterone levels were flattened by implanting mice with subcutaneous corticosterone-releasing pellets, resulting in constant circulating corticosterone levels.

**Results:**

Flattened corticosterone rhythm caused a complete loss of circadian rhythm in triglyceride-derived fatty acid uptake by BAT. This effect was independent of glucocorticoid receptor expression in (brown) adipocytes and was not caused by deregulation of clock gene expression or overexposure to glucocorticoids, but rather seemed mediated by reduced sympathetic innervation of BAT. In a mouse model of hyperlipidemia and metabolic syndrome, long-term experimental flattening of corticosterone − and thus rhythm in BAT function − resulted in adiposity.

**Conclusions:**

This study highlights that a physiological rhythm in glucocorticoids is an important regulator of BAT function and essential for the maintenance of metabolic health.

## Highlights

• Flattening of corticosterone rhythm blunts circadian activity of brown adipose tissue.
• Disturbed corticosterone rhythm − rather than overexposure− is responsible for blunted brown adipose tissue activity.
• The metabolic effect of flattened corticosterone levels is independent of adipocyte glucocorticoid receptor expression.
• Long-term flattening of corticosterone levels results in increased adiposity in a female mouse model for metabolic syndrome.

## 1 Introduction

Brown adipose tissue (BAT) is a highly metabolically active tissue in human adults [1]. The mitochondria of brown adipocytes express uncoupling protein 1 (UCP1), which dissipates the proton gradient across the inner membrane that is normally necessary for production of adenosine triphosphate (ATP) [2]. Instead, heat is generated in a process called non-shivering thermogenesis. Thermogenesis utilizes intracellular lipids as fuel [3], and these lipids are replenished by the uptake of triglyceride (TG)-derived fatty acids (FAs). This process is dependent on lipoprotein lipase (LPL), which hydrolyzes TG from lipoproteins [4,5]. Due to its ability to convert biochemical energy into heat, BAT has emerged as a promising target to reduce adiposity [6]. BAT activity is regulated by the sympathetic nervous system, for example upon cold exposure. In mice, sympathetic nerve endings release norepinephrine (NE) to stimulate the β3-adrenergic receptors (β3AR) on brown adipocytes. Second messenger cyclic AMP (cAMP) subsequently promotes the activation of protein kinase A, which in turn stimulates lipolysis through hormone-sensitive lipase, as well as the expression of key thermogenic genes, such as *Ucp1* and *Pgc1a* via cAMP response element-binding protein (CREB) [6].

Previous studies have shown that BAT activity is regulated by the biological clock [[7], [8], [9], [10]]. The central biological clock, which is located in the suprachiasmatic nucleus (SCN) of the hypothalamus, receives light signals from the retina and subsequently synchronizes circadian (i.e., 24-h) rhythms in various tissues throughout the body. The SCN communicates with these tissues through the autonomic nervous system and endocrine signals [11]. Disturbances of the biological clock in humans, e.g., through shift work or artificial light exposure, are associated with an increased risk of obesity, type 2 diabetes, and dyslipidemia [12], and studies in rodents suggest a causal relationship [13]. BAT may underlie the relationship between rhythm disturbances and metabolic disease, as BAT exhibits a strong diurnal rhythm in both mice [8,9] and humans [14]. Disruption of this rhythm by prolonged light exposure or genetic alternation in clock genes reduces the metabolic activity of BAT, resulting in increased adiposity in mice [7]. The exact mechanism by which the SCN transfers diurnal information to BAT is not fully understood. Further understanding of circadian BAT activity may benefit individuals with increased risk of metabolic disorders caused by a disruption of circadian rhythms.

Glucocorticoid hormones, such as corticosterone in rodents, are possible candidates for transferring the circadian time-keeping signal to BAT. By acting through the glucocorticoid receptor (GR), glucocorticoids entrain rhythms in several peripheral tissues [15,16], although it is unclear whether this is also the case for BAT. In this study, we report that glucocorticoid rhythm regulates circadian BAT activity in mice as measured by plasma TG-derived FA uptake, and that this rhythm is independent of GR expression in adipocytes. Furthermore, we demonstrate that disruption of this glucocorticoid rhythm – and thereby BAT activity – aggravates adiposity in mice.

## 2 Materials and methods

### 2.1 Animal experiments

All animal experiments were performed in accordance with the Institute for Laboratory Animal Research Guide for the Care and Use of Laboratory Animals after having received approval from the central animal experiments committee. All mice were housed on a 12-h:12-h light:dark cycle (07h00 lights on; 19h00 lights off) and under mild cold stress at 21 °C. Mice were fed *ad libitum*. Experiments were performed in 8- to 12-week-old male and female wild-type (WT; C57Bl/6J background) mice (Charles River Laboratories), 10- to 18-week-old male adipocyte-specific GR-deficient mice (ad.GRKO; Nr3c1^tm2Gsc^Tg (Adipoq-cre)1Evdr: GR^ΔAdip^; C57Bl/6 background; breeding University of Ulm) [17] and 12-to 18-week-old female APOE∗3-Leiden.CETP mice (C57Bl/6J background; breeding Leiden University Medical Center) [18]. WT and ad.GRKO mice were fed a standard chow diet. APOE∗3-Leiden.CETP mice were fed a Western-type diet containing 15% fat from cocoa butter and 1% fat from corn oil (diet T, Altromin) and enriched with 0.1% cholesterol.

Male WT mice were subcutaneously implanted in the neck region in between both interscapular BAT (iBAT) depots (approximately 5 mm distance from both) with a pellet containing either vehicle (100 mg of cholesterol) or 2.5 mg of corticosterone (2.5 mg of corticosterone and 97.5 mg of cholesterol, 100 mg in total; N = 16/group). Corticosterone release from the pellets results in constant corticosterone levels due to diminished endogenous corticosterone secretion at the time of the circadian peak, via increased negative feedback via the central high affinity mineralocorticoid receptor [19]. After 7 days of pellet exposure, food was removed at 02h00 or 14h00, and the uptake of TG-derived FAs by various adipose tissue depots was determined respectively between 06h00 or 08h00 (AM) and between 18h00 and 20h00 (PM) (N = 8/timepoint/group).

To study the importance of adipocyte GR, we utilized ad.GRKO mice and WT littermates (adipoq-cre negative). In a first cohort, male ad.GRKO mice (N = 13) and WT littermates (N = 14) were fasted for 8 h, and the uptake of TG-derived FAs was determined at AM (lights on) and PM (lights off) (N = 6–8/timepoint/genotype). In a second cohort, male ad.GRKO mice (N = 11) and WT littermates (N = 11) were subcutaneously implanted in the neck region with a pellet containing 2.5 mg of corticosterone. After 7 days of pellet exposure, food was removed at 23h00 or 11h00, and the uptake of TG-derived FAs by various adipose tissue depots was determined respectively between 06h00 and 08h00 (AM) or between 18h00 and 20h00 (PM) (N = 5–6/timepoint/genotype).

Female WT mice were subcutaneously implanted in the neck region with a pellet containing either 7.5 mg of corticosterone or vehicle (100 mg of cholesterol) and were subcutaneously injected daily with 3 mg/kg of corticosterone (corticosterone-HBC complex, Sigma–Aldrich C174; resulting in corticosterone levels that greatly exceeds the normal circadian peak [20]) or solvent 1 h before the mice were killed. After 7 weeks of pellet exposure, food was removed at 14h00, and the uptake of TG-derived FAs was determined between 18h00 and 20h00 (PM) (N = 10/group).

Female APOE∗3-Leiden.CETP mice were fed a run-in Western-type diet for 3 weeks, after which they were block randomized into experimental groups that were balanced for plasma TGs, plasma total cholesterol, body weight, and age. Mice were subcutaneously implanted in the neck region with either a 3.75-mg corticosterone pellet, 7.5-mg corticosterone pellet, or vehicle pellet (N = 8/group). Pellets were replaced every 2 weeks, and adequate flattening of the glucocorticoid rhythm by corticosterone-releasing pellets was confirmed by measuring plasma corticosterone. Three APOE∗3-Leiden.CETP mice from the 3.75-mg corticosterone-pellet group were excluded from the analysis, as corticosterone levels did not flatten following pellet implantation (defined by a rhythm amplitude with less than one SD difference as compared to the rhythm amplitude in the vehicle control group). Body weight, food intake, and body composition (EchoMRI-100-analyzer; EchoMRI) were determined throughout the experiment. After 5 weeks of pellet exposure, food was removed at 14h00, and the uptake of TG-derived FAs was determined between 18h00 and 20h00 (PM).

### 2.2 Indirect calorimetry

For measurements of energy expenditure, respiratory exchange ratio, physical activity, core body temperature, and food intake, mice were single-housed in metabolic home cages (Phenomaster, TSE Systems). After 3 days of acclimatization, measurements started at 20-min interval for a total of 7 days.

### 2.3 Plasma clearance and organ uptake of radiolabeled TG-derived FAs

TG-rich lipoprotein (TRL)-like emulsion particles (average size 80 nm) radiolabeled with glycerol tri [^3^H]oleate were prepared as previously described [4]. Mice were fasted for 4–8 h and injected intravenously with particles containing 1.0 mg of TG. Blood was collected by a nick in the tail vein at 2, 5, 10, and 15 min after injection in order to monitor plasma decay of the radiolabel. After 15 min, mice were killed by cervical dislocation, perfused for 5 min with ice-cold phosphate-buffered saline (PBS), and several metabolically active tissues were harvested to measure the ^3^H activity by liquid scintillation to evaluate TG-derived FA uptake.

### 2.4 Norepinephrine measurement

NE levels were measured in subscapular BAT (sBAT) tissue using a commercially available enzyme-linked immunosorbent assay (ELISA) Kit (CatNo. BA E- 52000, LND) that was modified for tissue homogenates. Tissue samples were homogenized in a tissue homogenizer (Precellys) with ceramic beads in a special buffer to stabilize NE (1 mM EDTA, 4 mM Na2S2O5, 0.01 M HCl). BAT samples were homogenized with the tenfold amount of buffer and centrifuged for 10 min at 13,000 rpm at 0 °C. Thereafter, the infranatant, between the cell debris and lipid layer, was isolated and centrifuged for 4 min at 13,000 rpm at 0 °C, and the protein content of the lysate was measured using PierceTM BCA protein assay kit (23225, Thermo Scientific). Five micrograms of BAT in a maximum of 25 μl of buffer were applied to the NE Research ELISA (LDN) and measured according to the manufacturer's protocol using the EnSpire® Multimode Plate Reader.

### 2.5 Corticosterone measurement

To determine plasma corticosterone levels, blood was collected in a stress-minimized manner, i.e., via a nick in the tail vein and within 2 min before stress-induced corticosterone levels rise [21]. Corticosterone concentration was measured by ELISA (Corticosterone EIA, Immunodiagnostics) according to the manufacturer's protocol.

### 2.6 Cell culture

Murine immortalized preadipocytes were generated and cultured as previously described [22]. Pre-adipocytes were differentiated for 14–15 days. During the last 2 days of differentiation and during the experiments, cells were grown in basal culture medium containing hormone-deprived (charcoal-stripped) serum (Gibco). To investigate whether corticosterone (Sigma–Aldrich) can induce rhythm in brown adipocytes, cells were pretreated with or without 1 μM of GR antagonist RU486 (Sigma–Aldrich) for 30 min, followed by treatment with 10 nM of corticosterone or vehicle, and cells were harvested at T = 0, 8, 16, 24, or 32 h after treatment for gene expression analysis.

### 2.7 Gene expression analysis

Total mRNA was extracted from cultured brown adipocytes and iBAT by using TRIzol RNA isolation reagent (Thermo Fisher) following the manufacturer's protocol. RNA concentration was determined with a NanoDrop spectrophotometer (Thermo Fisher), and 1 μg of RNA was reverse transcribed to cDNA using M-MLV Reverse Transcriptase (Promega). Quantitative reverse transcription polymerase chain reaction (qRT-PCR) was performed using a SYBR Green kit (Promega) on a CFX96 PCR machine (Bio-Rad), and expression levels of genes of interest were normalized using a housekeeping gene. Primer sequences: *Lpl-*Fwd: CCCTAAGGACCCCTGAAGAC; *Lpl-*Rev: GGCCCGATACAACCAGTCTA; *Per2-*Fwd: TGTGTGCTTACACGGGTGTCCTA; *Per2*-Rev: *RevErbα*-Fwd: ACGTTTGGTTTGCGCATGAA; GTGCTTGTCTCTGCAGACCG; *RevErbα-*Rev: TTGGTGAAGCGGGAAGTCTC.

### 2.8 Protein isolation and western blot analysis

iBAT samples were homogenized and diluted in lysis buffer. Homogenates were centrifuged and protein content of the supernatant was determined using a BCA protein assay kit (Thermo Scientific). After heating the samples (5 min, 95 °C), 20 μg of protein was separated by 10% sodium dodecyl sulfate polyacrylamide gel electrophoresis (SDS-PAGE), followed by transfer to a nitrocellulose membrane. Membranes were blocked with 5% milk and incubated overnight at 4 °C with the primary antibody rabbit anti-pCREB S133 (Cell Signaling; 1:1,000), goat anti-LPL (in house antibody; 1:5,000), mouse anti-β-actin (Sigma; 1:1,000), or rabbit anti-GAPDH (Santa Cruz; 1:1,000), followed by incubation for 1 h with horseradish peroxidase (HRP)-conjugated secondary antibodies (anti-rabbit or anti-mouse; Promega at 1:5,000; anti-goat 1:20,000). Bands were visualized using SuperSignal Western Blot Enhancer (Thermo Scientific) and analyzed with a ChemiDoc Touch Imaging System (Bio-Rad). Protein expression of pCREB was normalized to that of the housekeeping protein β-actin and LPL to GAPDH.

### 2.9 Histological analysis

Adipose tissues (i.e., gonadal white adipose tissue (gWAT), subcutaneous WAT (sWAT), and iBAT) were fixated in 4% formalin, embedded in paraffin, and cut into 5-μm sections. Slides were stained with hematoxylin and eosin (H&E) using standard protocols. UCP1 and tyrosine hydroxylase (TH) staining was performed as previously described [7]. White adipocyte size, iBAT lipid droplet content, and UCP1/TH expression (relative UCP1 or TH staining per area) were quantified using ImageJ software (Version 1.50).

### 2.10 Statistical analysis

Data are presented as means ± SEM. Statistical analysis was performed using GraphPad Prism (version 7.02). Means were compared using one- or two-way analysis of variance (ANOVA) with Sidak or Dunnett's post hoc test, as indicated in figure legends. Differences between groups were considered statistically significant at p < 0.05.

## 3 Results

### 3.1 Flattening of corticosterone rhythm blunts diurnal TG-derived FA uptake by BAT *in vivo*

**Figure 1:**
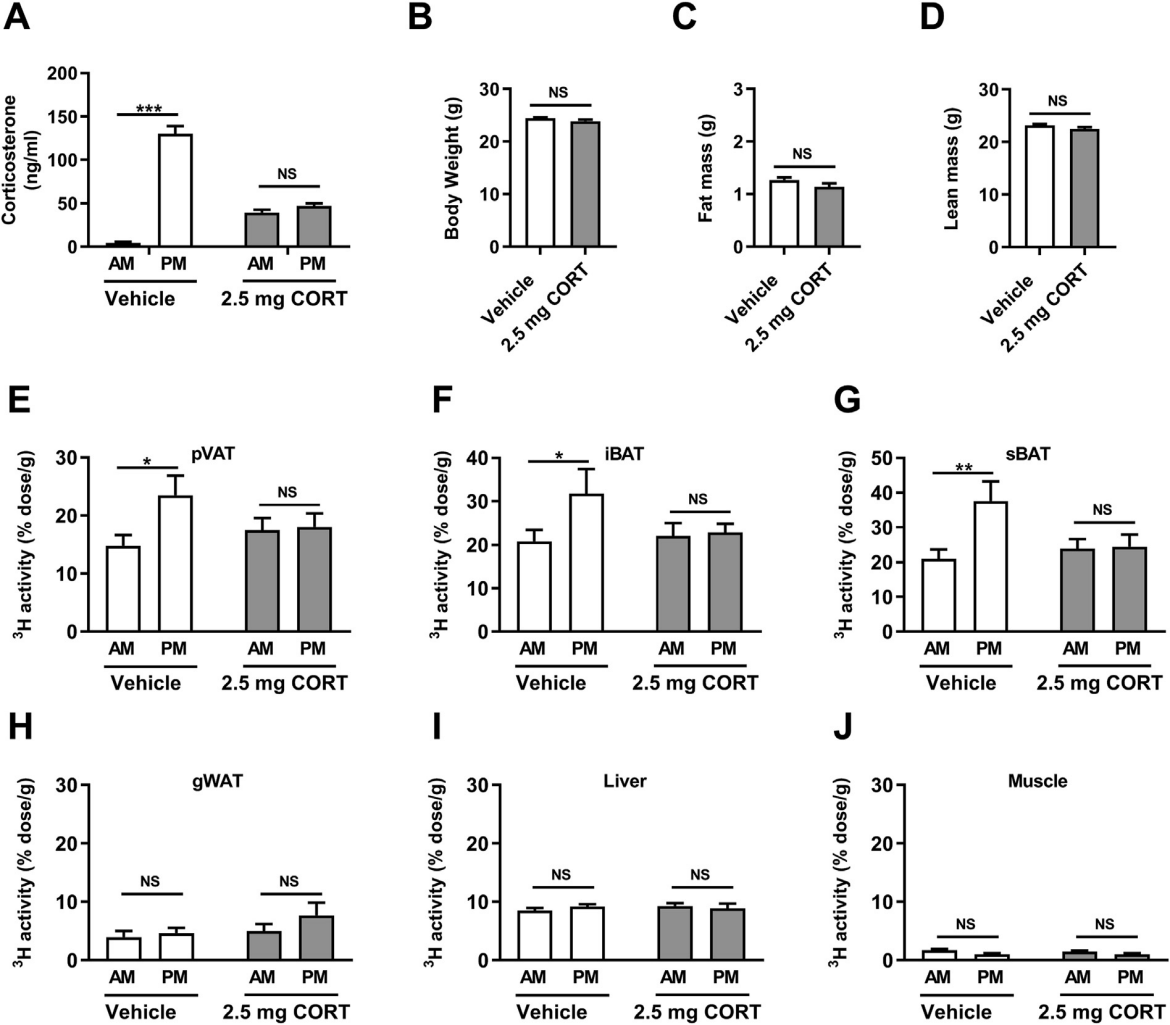
Flattening of the corticosterone rhythm blunts rhythmic BAT activity in wild-type mice. (A) Plasma corticosterone levels in male WT mice implanted with vehicle and 2.5-mg corticosterone (CORT) pellets at AM (lights on) and PM (lights off) of day 7. (B) Body weight, (C) fat mass, and (D) lean mass of vehicle- and corticosterone-treated mice at day 7. Uptake of [^3^H]oleate by (E) perivascular adipose tissue (pVAT), (F) interscapular brown adipose tissue (iBAT), (G) subscapular brown adipose tissue (sBAT), (H) gonadal white adipose tissue (gWAT), (I) liver, and (J) muscle at day 7. Data represent means ± SEM (N = 8/group/time point). ∗p < 0.05, ∗∗p < 0.01, ∗∗∗p < 0.001. NS: non-significant. Statistical difference was calculated using a two-way ANOVA with Sidak's multiple comparisons test (A, E-J) or by an independent student's t-test (B–D).

**Supplementary Figure 1:**
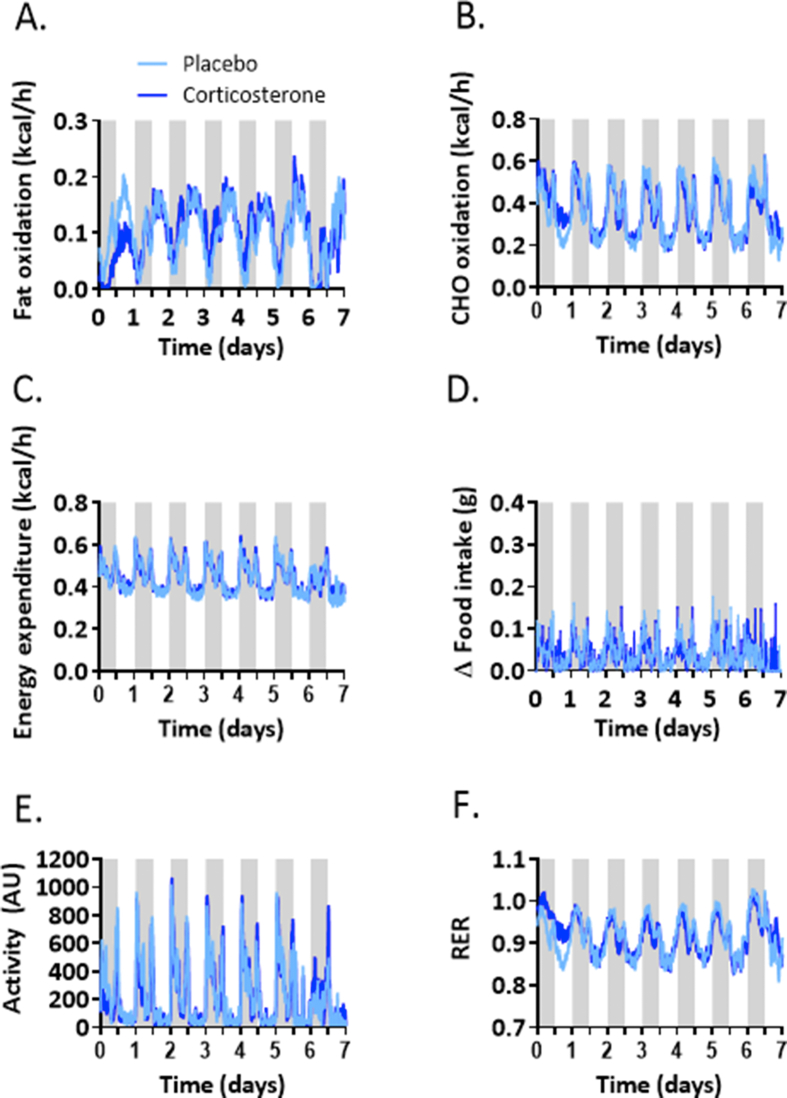
**Flattening of corticosterone rhythm does not influence energy expenditure in wild-type (WT) mice.** Metabolic cage measurements to determine (A) fat oxidation, (B) carbohydrate (CHO) oxidation, (C) energy expenditure, (D) food intake, (E) physical activity, and (F) respiratory exchange ratio (RER) in male WT mice implanted with placebo- and 2.5-mg corticosterone pellets.

**Supplementary Figure 2:**
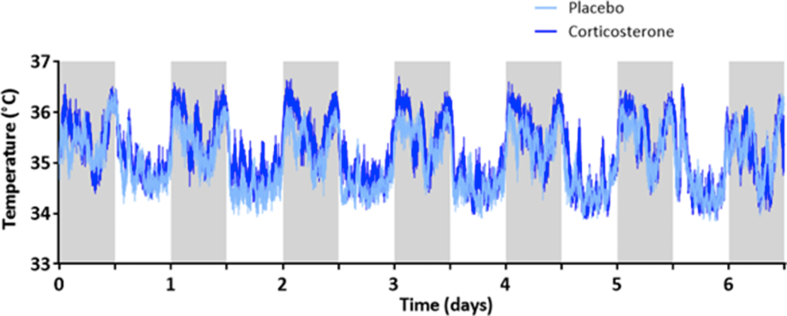
**Flattening of corticosterone rhythm does not influence body temperature and activity in wild-type (WT) mice.** Metabolic cage measurements to determine core body temperature in male WT mice implanted with placebo- and 2.5-mg corticosterone pellets.

**Supplementary Figure 3:**
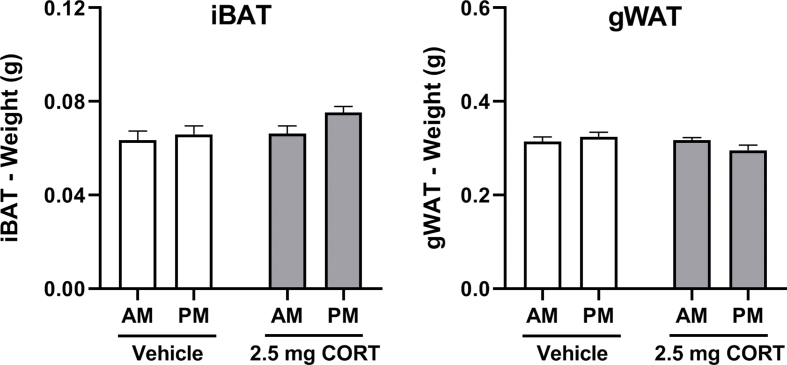
**Flattening of corticosterone rhythm does not influence iBAT and gWAT tissue weight in wild-type (WT) mice.** Interscapular brown adipose tissue (iBAT) and subscapular brown adipose tissue (sBAT) tissue weight in male WT mice implanted with vehicle- or 2.5-mg corticosterone (CORT) pellets for 7 days.

To investigate whether diurnal glucocorticoid rhythm and BAT activity are linked, we first implanted lean male WT mice with corticosterone-releasing pellets to evoke constant corticosterone levels throughout the day (Figure 1A). This was compared with control WT mice implanted with vehicle-pellets who exhibit an endogenous corticosterone rhythm with low circulating levels at AM and high circulating levels at PM (Figure 1A). Flattening of corticosterone did not influence body weight, total fat mass, and lean mass after 7 days of exposure (Figure 1B–D). Also, energy expenditure (Suppl. Fig. 1A–C), food intake (Suppl. Fig. 1D), physical activity (Suppl. Fig. 1E), and core body temperature (Suppl. Fig. 2), as well as the tissue weights of iBAT and gWAT (Suppl. Fig. 3) were unaltered by flattening of corticosterone in WT mice. In line with our previous report [8], control mice exhibited a robust rhythm in the uptake of TG-derived FAs by the thermogenic tissues perivascular adipose tissue (pVAT; Figure 1E), iBAT (Figure 1F), and sBAT (Figure 1G), with a higher uptake in the evening (PM) compared to the morning (AM). Strikingly, flattening of corticosterone levels completely abolished the rhythmic activity of these thermogenic tissues, as reflected by a similar FA uptake in thermogenic tissues at AM and PM (Figure 1E–G). More specifically, flattening of corticosterone prevented the increase in TG-derived FA uptake at PM. Other tissues, such as gWAT, liver, and muscle did not display a rhythm in the uptake of TG-derived FAs in control mice, and flattening of corticosterone did not affect FA uptake in these tissues (Figure 1H–J).

**Figure 2:**
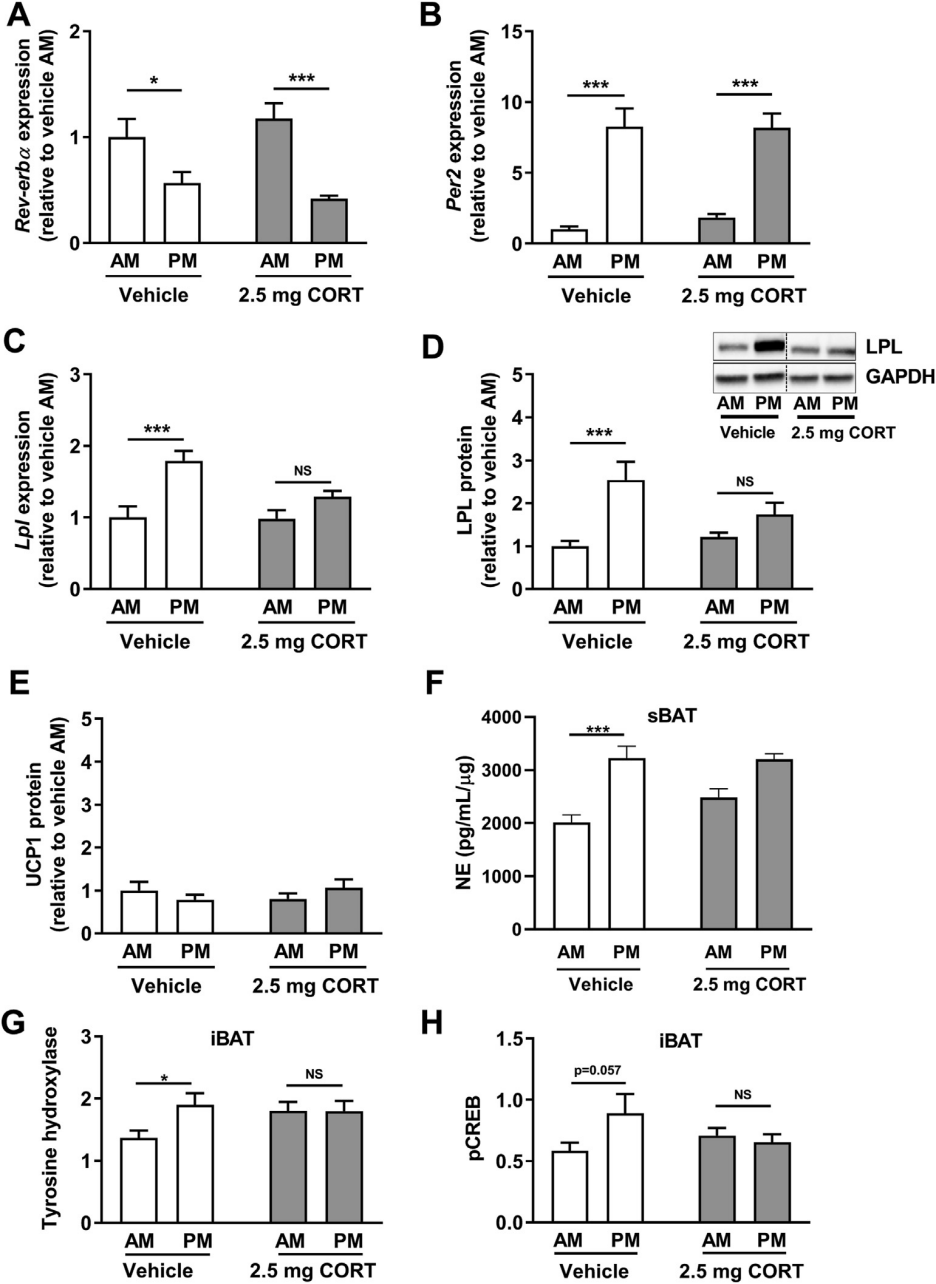
Flattening of corticosterone rhythm modulates LPL expression and adrenergic signaling in BAT of wild-type mice. mRNA expression of the genes (A) *Rev-erbα*, (B) *Per2,* and (C) *Lpl* and (D) protein expression of LPL was determined in interscapular brown adipose tissue (iBAT) of WT mice implanted with vehicle or 2.5-mg corticosterone (CORT) pellets at AM (lights on) and PM (lights off) of day 7. Data represents means ± SEM (N = 8/group/timepoint). ∗p < 0.05, ∗∗∗p < 0.001. Statistical significance was calculated using a two-way ANOVA with Sidak's multiple comparisons test. p^interaction^ = 0.069 for *Lpl* mRNA and 0.026 for LPL protein. (E) UCP1 protein expression in iBAT, (F) norepinephrine (NE) in sBAT, (G) tyrosine hydroxylase (TH) staining in iBAT and (H) phosphorylated CREB (pCREB) protein in iBAT of male WT mice implanted with vehicle or 2.5-mg corticosterone (CORT) pellets, at AM (lights on) and PM (lights off) of day 7. Data represent means ± SEM (N = 3–8/group/timepoint). ∗p < 0.05, ∗∗∗p < 0.001. NS: non-significant. Statistical significance was calculated using a two-way ANOVA with Sidak's multiple comparisons test. p^interaction^ = 0.035 for TH and 0.061 for pCREB.

In BAT obtained from lean male control WT mice, expression of clock genes was highly rhythmic with high *Rev-erba* expression at AM and low expression at PM (Figure 2A) and low *Per2* expression at AM and high expression at PM (Figure 2B). Flattening of corticosterone levels with corticosterone-pellets did not influence the rhythmicity of *Rev-erba* and *Per2* expression (Figure 2A–B). In line with the rhythm in TG-derived FA uptake by BAT, *Lpl* mRNA and LPL protein were both highly rhythmic in control mice, while they were blunted in mice with flattened corticosterone rhythm (Figure 2C–D). Protein expression of UCP1 was not rhythmic in iBAT and was unaffected by flattening of the corticosterone rhythm (Figure 2E). Tissue NE levels in BAT were rhythmic, while corticosterone flattening decreased its amplitude (difference between AM and PM; Figure 2F). In line with tissue NE levels, expression of tyrosine hydroxylase (TH), the rate-limiting enzyme for local NE synthesis, was also found to be rhythmic in BAT of control WT mice. TH expression was blunted in corticosterone-treated mice, as no difference was found between AM and PM (Figure 2G). In line with this observation, CREB phosphorylation, an important downstream event of NE-induced β3AR activity, was non-significantly higher at PM of control mice and not rhythmic in corticosterone-treated mice (Figure 2H).

### 3.2 Corticosterone induces rhythmic expression of molecular clock genes but not of genes involved in BAT activity

**Figure 3:**
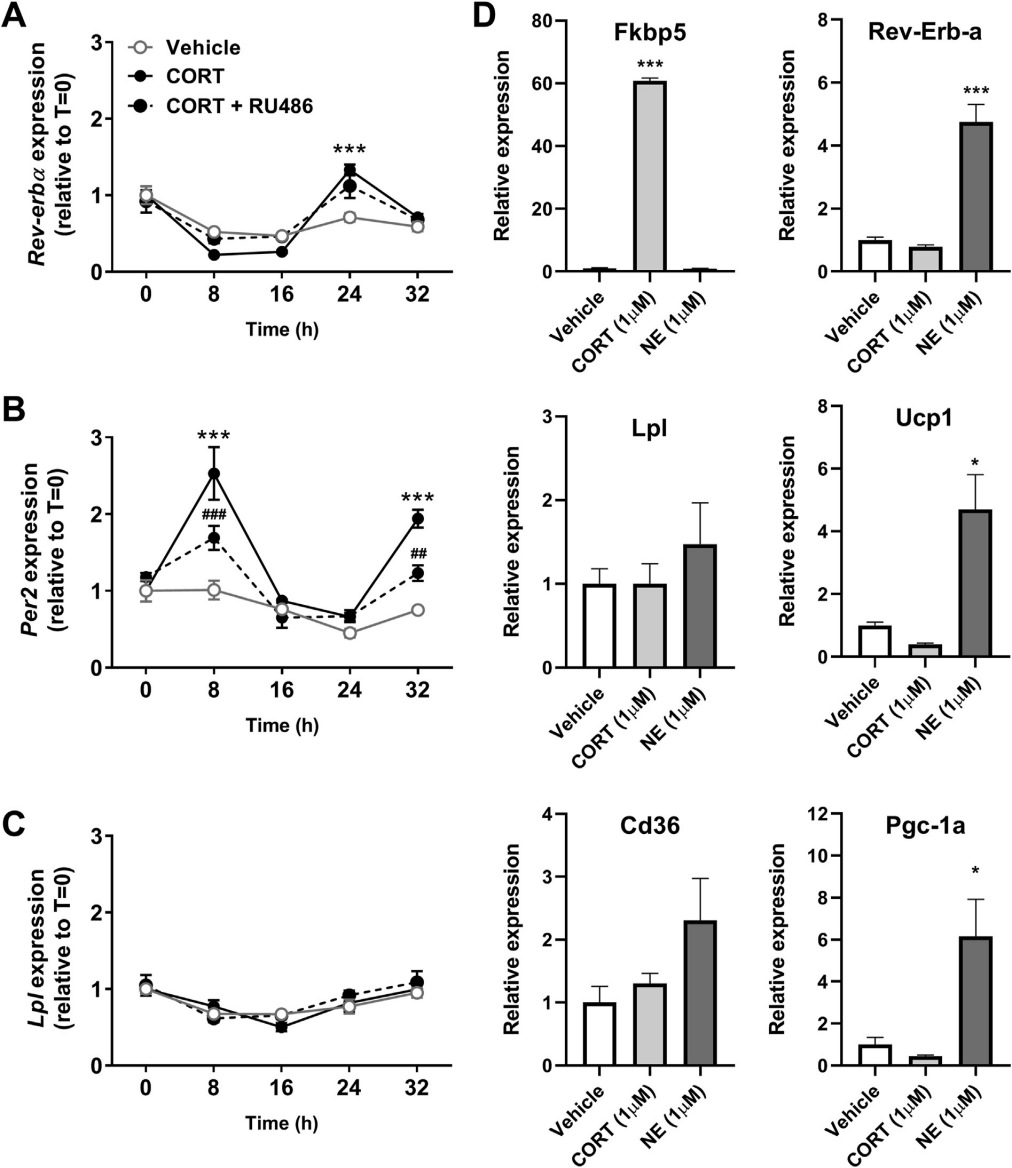
Corticosterone induces rhythmic expression of molecular clock genes but not *Lpl*. mRNA expression of the genes (A) *Rev-erbα*, (B) *Per2,* and (C) *Lpl* was determined in immortalized brown adipocytes treated with vehicle, corticosterone (CORT; 10 nM), or CORT with RU486 pretreatment (1 μM). Statistical significance was calculated using a two-way ANOVA with Sidak's multiple comparisons test. Data represent means ± SEM (N = 4/group/timepoint). ∗∗∗p < 0.001 compared to the vehicle, ^##^p < 0.01 compared to CORT, ^###^p < 0.001 compared to CORT. (D) mRNA expression of *Fkbp5*, *Rev-Erb-a*, *Lpl*, *Ucp1*, *Cd36*, *Pgc-1a* was determined in immortalized brown adipocytes treated with CORT (1 μM) or norepinephrine (NE, 1 μM). ∗p < 0.05 compared to vehicle, ∗∗∗p < 0.001 compared to vehicle.

Although previous studies have demonstrated direct involvement of clock genes in BAT function [10,23], our present study suggests that the effects of flattened corticosterone levels are not mediated through modulation of the intrinsic molecular clock in BAT (Figure 2A–B). To confirm this, we exposed cultured murine brown adipocytes to a low concentration of corticosterone (10 nM) and harvested cells every 8 h to evaluate rhythmic gene expression. As expected, corticosterone treatment synchronizes brown adipocytes, evidenced by a robust rhythm in the expression of circadian clock genes *Rev-erba* (Figure 3A) and *Per2* (Figure 3B). This process was at least partly GR-dependent, as pretreatment with the GR antagonist RU486 attenuated rhythmic gene expression of clock genes (Figure 3A–B). However, corticosterone treatment of brown adipocytes did not induce rhythmic expression in *Lpl* (Figure 3C) or other genes involved in metabolic BAT activity, such as *Ucp1* (data not shown). To investigate whether the blunted BAT rhythm *in vivo* is mediated via NE signaling, we evaluated the direct effect of NE treatment on cultured brown adipocytes. This revealed that NE treatment significantly increased the expression of *Reb-Erb-a*, *Ucp1* and *Pgc-1a*, while it non-significantly increased expression of *Lpl* and *Cd36* (Figure 3D). This shows that NE directly influences brown adipocyte expression of genes involved in the molecular clock and lipid uptake.

### 3.3 Adipocyte GR expression is dispensable for circadian TG-derived FA uptake by BAT

**Figure 4:**
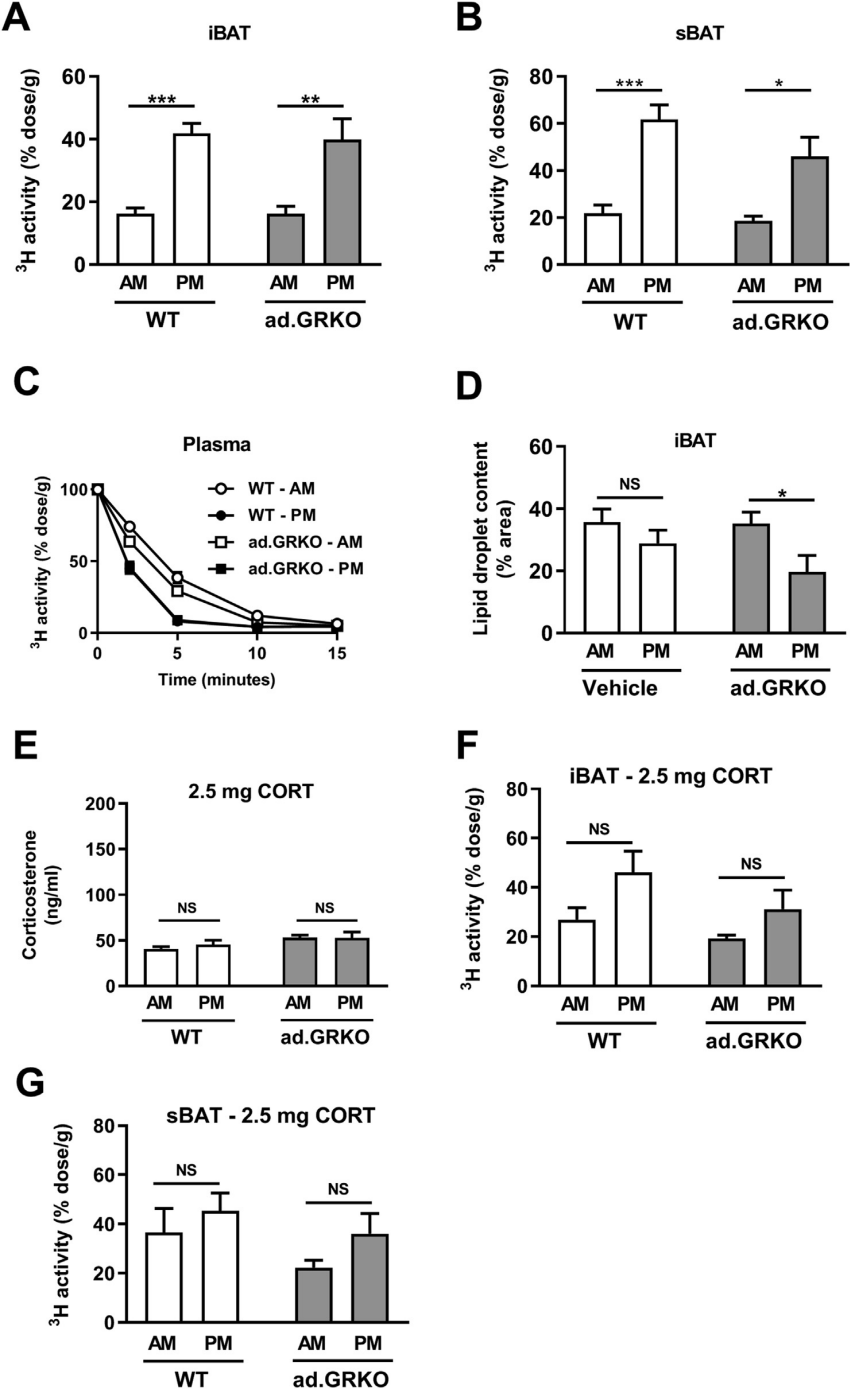
Adipocyte GR is dispensable for circadian BAT activity. Uptake of [^3^H]oleate in (A) interscapular brown adipose tissue (iBAT) and (A) subscapular brown adipose tissue (sBAT) of male wild-type (WT) and adipocyte-specific GR-deficient (ad.GRKO) mice at AM (lights on) and PM (lights off). (C) Plasma decay of [^3^H]oleate at AM (lights on) and PM (lights off). (D) Lipid droplet content of iBAT. (E) Corticosterone levels in mice implanted with 2.5-mg corticosterone (CORT) pellets at AM (lights on) and PM (lights off). Uptake of [^3^H]oleate in (F) iBAT and (G) sBAT of male WT and ad.GRKO mice implanted with 2.5-mg CORT pellets, at AM (lights on) and PM (lights off) of day 7. Data represent means ± SEM (N = 5–8/group/timepoint). ∗p < 0.05, ∗∗p < 0.01, ∗∗∗p < 0.001. NS: non-significant. Statistical significance was calculated using a two-way ANOVA with Sidak's multiple comparisons test.

As BAT function is affected by supraphysiologic levels of corticosterone [[24], [25], [26]], we continued by investigating whether the GR on (brown) adipocytes could mediate circadian BAT activity through other mechanisms than synchronizing the intrinsic molecular clock. To this end, we compared mice that lack GR in adipocytes (ad.GRKO mice) with WT littermates. To determine BAT rhythm in these mice, we evaluated the uptake of TG-derived FAs, which revealed a robust rhythm of TG-derived FA uptake in iBAT (Figure 4A) and sBAT (Figure 4B) of both WT and ad.GRKO mice, without differences between genotypes. Both genotypes displayed a similar plasma decay of TGs, with a faster clearance at PM as compared to AM (Figure 4C). Comparable lipid droplet areas were observed in iBAT of WT and ad.GRKO mice, with lower lipid content in ad.GRKO mice at PM when BAT is more active (Figure 4D). In a second cohort of ad.GRKO mice and WT littermates, we flattened corticosterone levels with corticosterone-releasing pellets (Figure 4E). Flattened corticosterone disrupted BAT rhythm similarly in WT and ad.GRKO mice, with no significant differences between AM and PM in TG-derived FA uptake in iBAT and sBAT (Figure 4F–G). These data demonstrate that adipocyte GR is dispensable for BAT activity rhythm and that corticosterone-induced flattening of rhythm in FA uptake is independent of adipocyte GR expression.

### 3.4 Overexposure to corticosterone does not reduce TG-derived FA uptake by BAT

**Figure 5:**
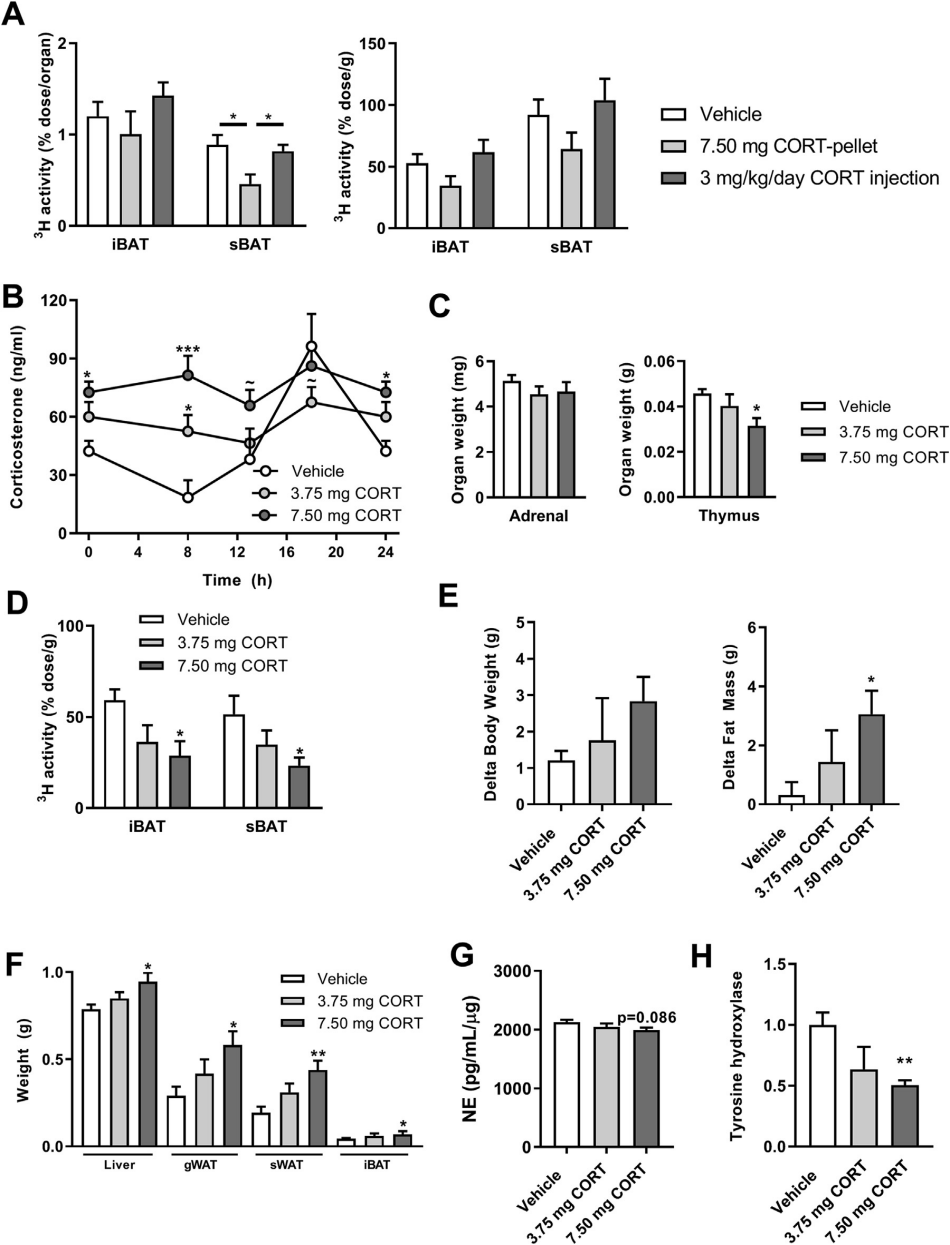
Flattening of the corticosterone rhythm decreases BAT activity and increases adiposity in APOE∗3-Leiden.CETP mice. (A) Uptake of [^3^H]oleate at PM (lights off) of day 49 in interscapular brown adipose tissue (iBAT) and subscapular brown adipose tissue (sBAT) per g tissue or per total organ, in female WT mice implanted with vehicle or 7.5-mg corticosterone (CORT) pellets or upon daily injection with 3 mg/kg/day corticosterone at PM. (B) Plasma corticosterone levels and area under the curve (AUC) of female APOE∗3-Leiden.CETP mice implanted with vehicle, 3.75-mg or 7.5-mg CORT pellets at day 7. (C) Weight of the left adrenal and thymus. (D) Uptake of [^3^H]oleate at PM (lights off) in iBAT and sBAT at day 35. (E) Delta body weight and delta fat mass over a period of 35 days. (F) Tissue weights of liver, gonadal white adipose tissue (gWAT), iBAT, and sBAT. (G) Norepinephrine (NE) levels in sBAT. (H) tyrosine hydroxylase (TH) staining in iBAT. Data represent means ± SEM (N = 5–8/group). ∗p < 0.05, ∗∗p < 0.01, ∗∗∗p < 0.001 compared to the vehicle group. Statistical significance was calculated using a one-way ANOVA with Tukey's multiple comparisons test (AUC of B; A, C-G) or a two-way ANOVA (B) with Dunnett's post hoc test.

In male WT mice, we did not observe short-term effects of corticosterone flattening on body weight and composition after 7 days, despite reduced BAT activity (Figure 1A–D). We next evaluated how flattening of corticosterone influences BAT activity and metabolic health in female WT mice. Based on a pilot experiment (data not shown), we established that the corticosterone dose required to generate a flattened rhythm in female mice is higher (7.5 mg) than that in male mice (2.5 mg), most likely due to the higher levels of corticosteroid-binding globulin in females [27]. Flattening of corticosterone rhythm with 7.5-mg corticosterone-pellets in female mice reduced TG-derived FA uptake by sBAT at PM (Figure 5A), without influencing the total fat mass over a period of 7 weeks (data not shown), similar to the effects observed in male mice. To confirm that the reduced BAT activity was due to a loss of corticosterone rhythm, rather than overexposure to corticosterone, we evaluated the effect of flattened corticosterone rhythm (corticosterone-pellets) versus mice injected daily with a high dose of 3 mg/kg of corticosterone at PM resulting in a 5-fold increase in peak corticosterone levels [20]. In contrast to mice with corticosterone-pellets, high-dose corticosterone injection did not influence TG-derived FA uptake by BAT at PM (Figure 5A). This suggests that flattened corticosterone rhythm and not overexposure to corticosterone is responsible for the decreased BAT activity.

### 3.5 Flattening of corticosterone rhythm causes adiposity in APOE∗3.Leiden-CETP mice

We next performed another long-term experiment in hyperlipidemic female APOE∗3.Leiden-CETP mice, which are mice with a humanized lipid metabolism that are prone to developing metabolic disease [18]. Herein, we investigated whether flattened corticosterone and perturbed BAT activity rhythm caused metabolic disturbances. Western-type diet-fed female APOE∗3.Leiden-CETP mice were exposed to low- (3.75 mg) or high (7.5 mg)-dose corticosterone-releasing pellets for 5 weeks. To estimate the total corticosterone exposure, we collected blood at various times throughout the day and night, which revealed constant, non-rhythmic plasma levels of corticosterone in mice treated with both low- and high-dose corticosterone pellets (Figure 5B). Both doses increased the area under the curve (AUC), reflecting a higher total corticosterone exposure, but corticosterone levels did not exceed the peak value in corticosterone of the vehicle-treated mice (Figure 5B). Low-dose corticosterone pellets did not alter the weight of the glucocorticoid-sensitive adrenal and thymus, suggesting no overt overexposure to corticosterone, while high-dose corticosterone slightly decreased thymus weight (Figure 5C). Along with a flattened corticosterone rhythm, we found a dose-dependent reduction in the uptake of TG-derived FAs in both iBAT and sBAT of corticosterone-treated mice (Figure 5D). In line with a decreased BAT activity, corticosterone exposure resulted in increased body fat gain (Figure 5E) and increased weights of liver, gWAT, sWAT, and iBAT (Figure 5F). Exposure to 7.5 mg of corticosterone non-significantly lowered tissue NE levels in sBAT (p = 0.086, Figure 5G) and significantly lowered TH expression in iBAT (Figure 5H).

**Figure 6:**
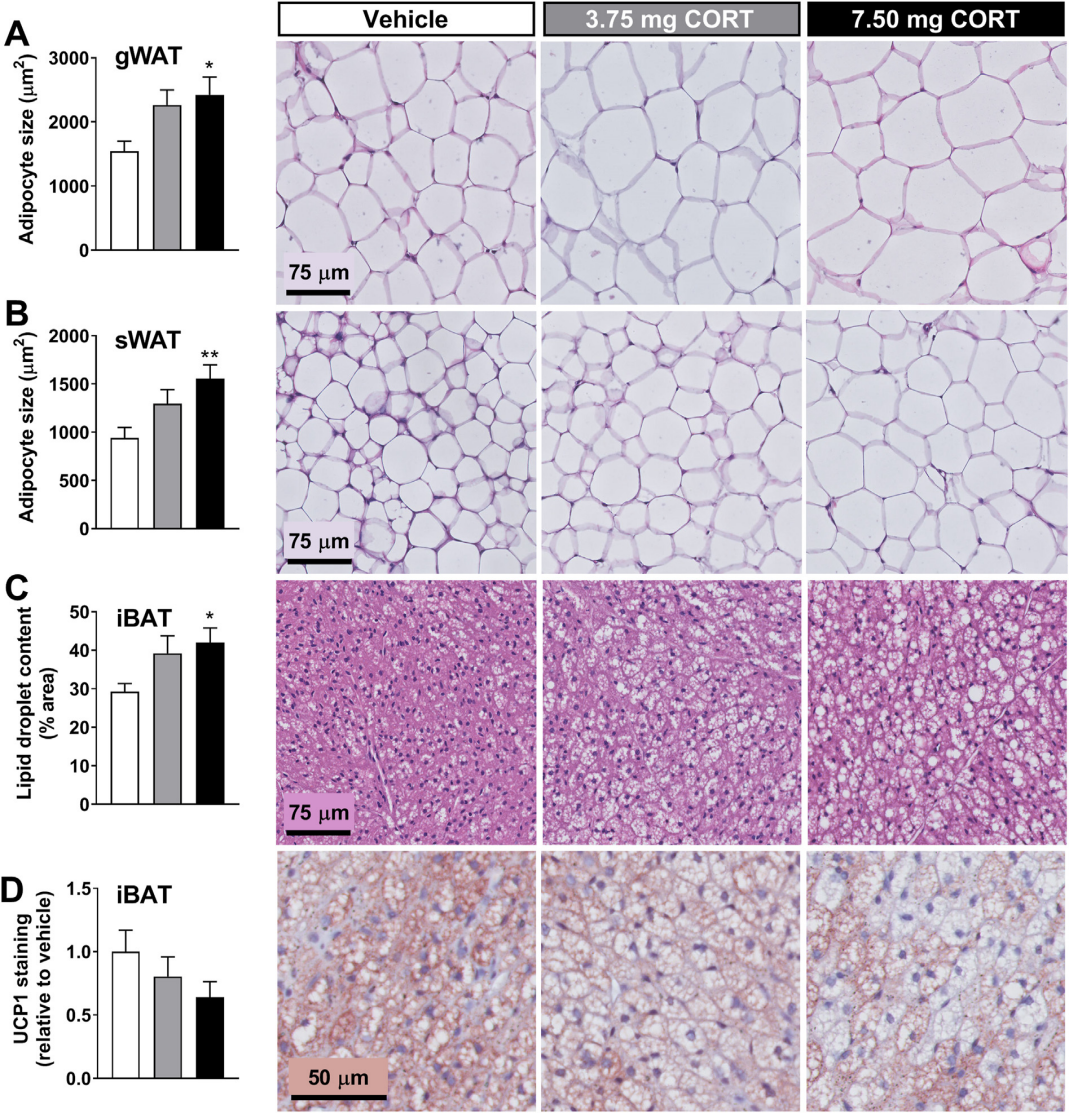
Flattening of corticosterone rhythm induces lipid accumulation in adipose tissue of APOE∗3-Leiden.CETP mice. Adipocyte size based on hematoxylin and eosin (H&E) staining in (A) gonadal white adipose tissue (gWAT) and (B) subcutaneous white adipose tissue (sWAT) from female APOE∗3-Leiden.CETP mice implanted with vehicle, 3.75-mg or 7.5-mg corticosterone (CORT) pellets. (C) Lipid content, (D) uncoupling protein-1 (UCP1) expression. Data represent means ± SEM (N = 5–8/group). ∗p < 0.05, ∗∗p < 0.01 compared to the vehicle control group. Statistical significance was calculated using a one-way ANOVA with Tukey's multiple comparisons test.

**Supplementary Figure 4:**
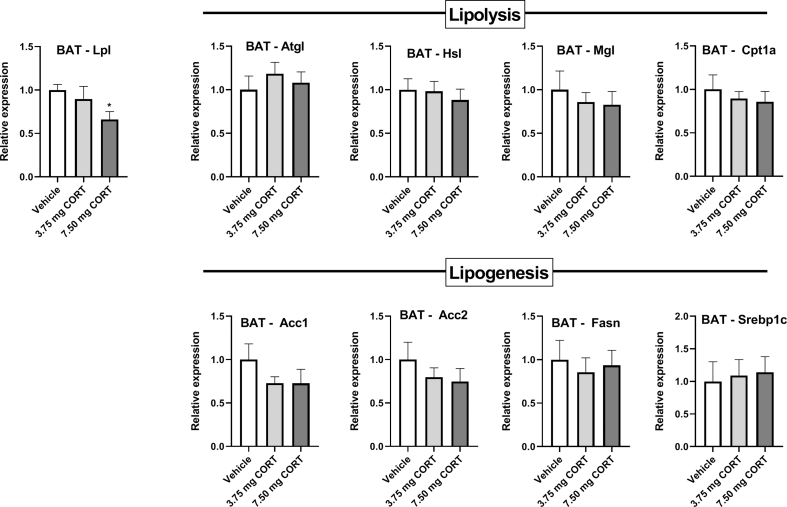
**Flattening of corticosterone rhythm decreases Lpl expression in iBAT.** mRNA expression of lipoprotein lipase (*Lpl*) and genes involved in lipolysis and lipogenesis in interscapular brown adipose tissue (iBAT) of female APOE∗3-Leiden.CETP mice implanted with vehicle, 3.75-mg or 7.5-mg corticosterone (CORT) pellets.

**Supplementary Figure 5:**
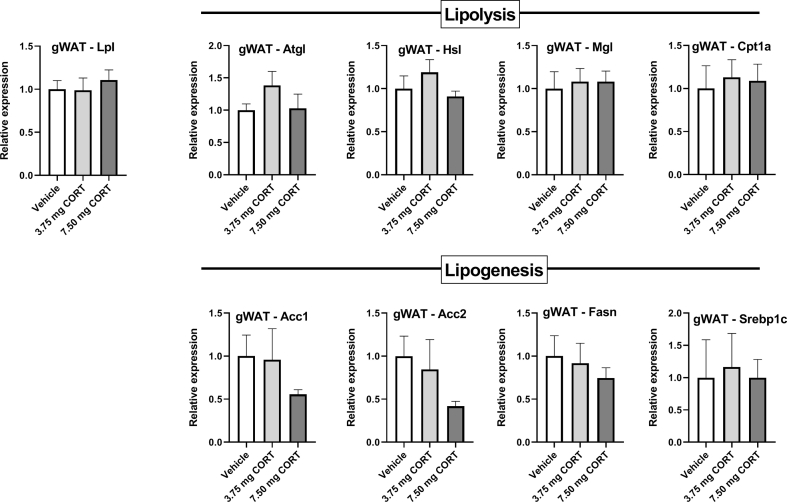
**Flattening of corticosterone rhythm does not influence expression of genes involved in lipolysis and lipogenesis in gWAT.** mRNA expression of lipoprotein lipase (*Lpl*) and genes involved in lipolysis and lipogenesis in gonadal white adipose tissue (gWAT) of female APOE∗3-Leiden.CETP mice implanted with vehicle, 3.75-mg or 7.5-mg corticosterone (CORT) pellets.

Increased tissue weights are likely attributed to enhanced lipid deposition, as corticosterone pellets increased adipocyte sizes of gWAT (Figure 6A) and sWAT (Figure 6B) and increased iBAT lipid droplet content (Figure 6C). Corticosterone exposure tended to reduce UCP1 expression (Figure 6D), and, together with the reduced local NE levels and the diminished TH expression in BAT (Figure 5G–H), this is consistent with a decreased BAT activity due to impaired sympathetic innervation. In line with previous experiments in male WT mice, corticosterone flattening reduced *Lpl* expression in iBAT at PM (Suppl. Fig. 4), while lipolysis and lipogenesis pathways were unaffected in both iBAT and gWAT (Suppl. Fig. 4-5).

## 4 Discussion

We and others have previously reported that BAT displays a robust circadian rhythm in metabolic activity [[8], [9], [10]], which coincides with circadian rhythm in plasma corticosterone in mice [8,28]. In the current study, we investigated the importance of corticosterone rhythm for circadian BAT activity and metabolic health. Our main findings demonstrate that flattening of the corticosterone rhythm blunts the rhythmicity in metabolic BAT activity by preventing the rise in activity at PM, and results in metabolic disturbances in mice. To our knowledge, we are the first to demonstrate that corticosterone rhythm regulates BAT activity rhythm, which we consistently show in males and females and across different mouse models. Our observations that corticosterone flattening causes adiposity in Western-type diet-fed APOE∗3.Leiden-CETP mice but not in chow-fed WT mice suggest that disturbance of BAT rhythm can cause metabolic symptoms depending on metabolic context.

We observed that flattened BAT activity is mirrored by a flattened rhythm in LPL expression. Hence, flattening of LPL expression likely explains flattened TG-derived FA uptake [8]. Our findings in cultured brown adipocytes demonstrate that corticosterone does not directly regulate *Lpl* expression. In line with this, GR activity in adipose tissue was also not essential for maintaining BAT activity rhythm *in vivo*, as mice that lack adipocyte GR expression still exhibit the robust rhythm in BAT metabolic activity as observed in WT mice. In accordance, a recent study showed that the GR in brown adipocytes is not required for BAT-dependent energy homeostasis [29]. Corticosterone flattening also perturbed BAT activity rhythm in ad.GRKO mice, indicating that this effect is also independent on direct binding of corticosterone to the GR on brown adipocytes. It is possible that the mineralocorticoid receptor on brown adipocytes is involved [30], but our *in vitro* data suggest that the rhythmic expression of *Lpl* is not cell-autonomous. Therefore, it is likely that corticosterone flattening blunts the rhythm in metabolic activity of BAT via pathways other than direct interaction with brown adipocytes.

In the present study, we show blunted rhythm in TH, NE, and phosphorylated CREB upon corticosterone flattening, which suggest that decreased sympathetic outflow may underlie blunted BAT rhythm. Indeed, glucocorticoids are known to pass the blood–brain barrier, where they can act on corticosteroid receptors within the central nervous system [31], and via this route, glucocorticoids have been shown to fine-tune sympathetic innervation of peripheral tissues, including BAT [[32], [33], [34]]. We previously showed that sympathetic denervation diminishes BAT rhythm [8], demonstrating that sympathetic innervation is essential for maintaining BAT rhythm. In the context of flattened glucocorticoid rhythm, our data indicates that blunted sympathetic innervation may be involved in decreased TG-derived FA uptake by BAT at PM; however, we cannot exclude that other processes may also modulate BAT function. For example, altered insulin signaling could influence (rhythmic) BAT function and could thereby contribute to the effects of corticosterone flattening. Indeed, previous studies show that corticosterone flattening, albeit at a higher total exposure, causes elevated plasma insulin levels and insulin resistance [35,36], but it is unclear whether this contributes to the altered TG-derived FA uptake in BAT.

A limitation of our study is the modest increase in total corticosterone exposure observed in mice implanted with corticosterone pellets. We cannot fully exclude that this increased corticosterone exposure may have contributed to the metabolic disturbances observed in these mice. Nevertheless, a recent study observed similar metabolic effects (i.e., increased body weight gain, fat accumulation in WAT and increased adipocyte size) in male mice with a disrupted glucocorticoid rhythm, while glucocorticoid excess timed at the right time of day (i.e., at the natural peak of glucocorticoids) did not further exacerbate metabolic symptoms [20]. We show similar findings in female WT mice, in which high-dose corticosterone treatment around the natural peak did not decrease BAT activity, while flattening of corticosterone rhythm did. These data corroborate our hypothesis that a disrupted glucocorticoid rhythm largely contributes to the adverse metabolic effects observed in subjects with excess glucocorticoid exposure. Another drawback of our study is the continuous negative feedback by the corticosterone-pellets on the hypothalamus-pituitary-adrenal axis. This negative feedback is required to blunt endogenous corticosterone production, but also diminishes endogenous adrenocorticotropic hormone (ACTH) secretion. It was previously shown that ACTH can directly activate BAT through cAMP signaling [24,37]. However, the effects of ACTH on BAT seem to occur only at supraphysiological levels [24], making this possibility unlikely. It should also be noted that throughout our study, we used TG-derived FA uptake as a proxy for BAT activity. Although we have shown that changes in FA uptake are typically accompanied by changes in thermogenic activity, also in the context of circadian rhythm, it may not necessarily reflect thermogenesis. Another limitation of our study is that we used male mice for the majority of experiments while only a subset of experiments was performed in females. For that reason, the results of our study cannot be broadly generalized to both sexes. Glucocorticoids are known to exhibit sexually dimorphic effects [38], and males are more sensitive to excess glucocorticoid exposure as compared to females [39,40]. From our studies, we cannot conclude whether the effects of corticosterone flattening (without excess exposure) also differentially influence BAT function in male and female mice. Finally, our experiments were performed under mild cold stress at 21 °C. At thermoneutrality, a condition with a different sympathetic tone, the effects of flattened corticosterone rhythm on BAT function could be different.

It is well established that patients with excess glucocorticoid exposure demonstrate profound metabolic disturbances as a result of endogenous glucocorticoid overproduction or due to therapeutic administration of exogenous glucocorticoids [41]. We show that flattened corticosterone rhythm within the physiological range also causes adiposity, suggesting that a lack of rhythm can significantly contribute to the development of metabolic symptoms in patients with perturbed endogenous glucocorticoid rhythm. It is important to note that species differences in glucocorticoid action on BAT have been reported [42]. Nevertheless, we believe that glucocorticoid rhythm likely plays a major role in regulating rhythmic BAT activity. Further research is required to fully elucidate the interplay between glucocorticoids, BAT rhythm, and metabolic health in humans.

In conclusion, we demonstrate that a physiological glucocorticoid rhythm is essential for rhythmic BAT function. We anticipate that disruption of glucocorticoid rhythm, and thereby BAT activity rhythm, could partially underlie the relationship between rhythm disturbances and metabolic disease in humans.

## Author contributions

Jan Kroon: Project administration, conceptualization, formal analysis, investigation, methodology, writing – original draft. Maaike Schilperoort: Project administration, conceptualization, formal analysis, investigation, methodology, writing – original draft. Wietse in het Panhuis: Investigation. Rosa van den Berg: Conceptualization, investigation. Lotte van Doeselaar: Investigation. Cristy R.C. Verzijl: Investigation. Nikki van Trigt: Investigation. Isabel M. Mol: Investigation. Hetty H.C.M. Sips: Investigation. Jose K. van den Heuvel: Methodology. Lisa L. Koorneef: Methodology. Ronald J. van der Sluis: Methodology. Anna Fenzl: Methodology. Florian W. Kiefer: Methodology. Sabine Vettorazzi: Methodology, resources. Jan P. Tuckermann: Methodology, resources. Nienke R. Biermasz: Funding acquisition. Onno C. Meijer: Supervision, writing – review and editing. Patrick C.N. Rensen: Funding acquisition, supervision, writing – review and editing. Sander Kooijman: Conceptualization, funding acquisition, supervision, writing – review and editing.
